# Rural and urban differences in stage at diagnosis of colorectal and lung cancers

**DOI:** 10.1054/bjoc.2000.1708

**Published:** 2001-04

**Authors:** N C Campbell, A M Elliott, L Sharp, L D Ritchie, J Cassidy, J Little

**Affiliations:** Department of General Practice and Primary Care, Foresterhill Health Centre, Westburn Road, Aberdeen, AB25 2AY; Department of Medicine and Therapeutics, Aberdeen University Medical School, Foresterhill, Aberdeen, AB25 2ZD

**Keywords:** colorectal cancer, lung cancer, epidemiology, rural, urban, staging

## Abstract

There is evidence that patients living in outlying areas have poorer survival from cancer. This study set out to investigate whether they have more advanced disease at diagnosis. Case notes of 1323 patients in north and northeast Scotland who were diagnosed with lung or colorectal cancer in 1995 or 1996 were reviewed. Of patients with lung cancer, 42% (69/164) living 58 km or more from a cancer centre had disseminated disease at diagnosis compared to 33% (71/215) living within 5 km. For colorectal cancer the respective figures were 24% (38/161) and 16% (31/193). For both cancers combined, the adjusted odds ratio for disseminated disease at diagnosis in furthest group compared to the closest group was 1.59 (*P* = 0.037). Of 198 patients with non-small-cell lung cancer in the closest group, 56 (28%) had limited disease (stage I or II) at diagnosis compared to 23 of 165 (14%) of the furthest group (*P* = 0.002). The respective figures for Dukes A and B colorectal cancer were 101 of 196 (52%) and 67 of 172 (39%) (*P* = 0.025). These findings suggest that patients who live remote from cities and the associated cancer centres have poorer chances of survival from lung or colorectal cancer because of more advanced disease at diagnosis. This needs to be taken into account when planning investigation and treatment services. © 2001 Cancer Research Campaign http://www.bjcancer.com


*British Journal of Cancer* (2001) **84**(7), 910–914
© 2001 Cancer Research Campaign
doi: 10.1054/ bjoc.2001.1708, available online at http://www.idealibrary.com on IDEAL®

http://www.bjcancer.com

### Rural and urban differences in stage at diagnosis of colorectal and lung cancers

**NC Campbell[1], AM Elliott[1], L Sharp[2], LD Ritchie[1], J Cassidy[3] and J Little[3]**

[1]Department of General Practice and Primary Care, Foresterhill Health Centre, Westburn Road, Aberdeen AB25 2AY; [2]Department of Medicine and Therapeutics, Aberdeen University Medical School, Foresterhill, Aberdeen, AB25 2ZD

**Summary** There is evidence that patients living in outlying areas have poorer survival from cancer. This study set out to investigate whether they have more advanced disease at diagnosis. Case notes of 1323 patients in north and northeast Scotland who were diagnosed with lung or colorectal cancer in 1995 or 1996 were reviewed. Of patients with lung cancer, 42% (69/164) living 58 km or more from a cancer centre had disseminated disease at diagnosis compared to 33% (71/215) living within 5 km. For colorectal cancer the respective figures were 24% (38/161) and 16% (31/193). For both cancers combined, the adjusted odds ratio for disseminated disease at diagnosis in furthest group compared to the closest group was 1.59 ($P$ = 0.037). Of 198 patients with non-small-cell lung cancer in the closest group, 56 (28%) had limited disease (stage I or II) at diagnosis compared to 23 of 165 (14%) of the furthest group ($P$ = 0.002). The respective figures for Dukes A and B colorectal cancer were 101 of 196 (52%) and 67 of 172 (39%) ($P$ = 0.025). These findings suggest that patients who live remote from cities and the associated cancer centres have poorer chances of survival from lung or colorectal cancer because of more advanced disease at diagnosis. This needs to be taken into account when planning investigation and treatment services. © 2001 Cancer Research Campaign http://www.bjcancer.com

**Keywords**: colorectal cancer; lung cancer; epidemiology; rural; urban; staging

In Scotland, colorectal and lung are the two most common cancers affecting both men and women (ISD, 1999). For both cancers, stage at diagnosis is a key indicator of prognosis. Overall 5-year survival for colorectal cancer is about 35% but this varies from 83% for tumours limited to the bowel wall (Dukes A) to 3% for tumours with distant metastases (SIGN, 1997; Summerton, 1999). The 30% of patients who present as emergencies fair worse than those presenting electively – 29% versus 39% 5-year survival (SIGN, 1997). For lung cancer, overall 5-year survival is only 6 or 7%, but again this varies considerably according to stage at diagnosis – there is 60 to 80% 5-year survival for stage I disease, but less than 5% 5-year survival for stage IIIb/IV (SIGN, 1998; Summerton, 1999).

Recognition of the benefits of specialized cancer care has led to the reshaping of cancer services in the United Kingdom (EAGC, 1995; Selby et al, 1996). There is a potential problem, however, that specialization may be accompanied by centralization and this has implications for about 20% of the United Kingdom population who live in rural areas. In France and the United States rural patients have been reported to have more advanced disease at diagnosis (Liff et al, 1991; Launoy et al, 1992), but there is limited research on this in the United Kingdom. In Scotland, outlying patients have been shown to have with less chance of diagnosis before death from common cancers (Campbell et al, 2000). This association was found using distance from cancer centres (which are located in the major centres of population) as the rural factor. It was not clear from the Scottish analysis, however, whether outlying patients who were alive when their cancer was diagnosed had more advanced disease than those resident in other areas because data on stage at diagnosis were not collected or analysed.

This study set out to investigate whether outlying patients had more advanced disease at diagnosis. The main hypotheses to be tested were that patients remote from cancer centres were more likely than those who lived nearby to present 1) with disseminated disease and 2) as emergencies.

*Received 25 July 2000*
*Revised 29 November 2000*
*Accepted 22 January 2001*

*Correspondence to:* Dr NC Campbell

### METHODS

### Setting

The study was set in north and northeast Scotland. About half the population and most cancer services were concentrated in Aberdeen and Inverness, each of which had a designated cancer centre (EAGC, 1995). At the time of the study, 5 smaller general hospitals (bed numbers ranging from 28 to 140) provided a variable amount of radiology, endoscopy and colorectal surgery (ISD, 1995). Most colorectal surgery and chemotherapy and all radiotherapy were conducted in Aberdeen and Inverness. All thoracic surgery was conducted in Aberdeen.

### Subjects

Data on all (1998) patients resident in Grampian and Highland Health Board areas and diagnosed with lung or colorectal cancers in 1995 and 1996 were obtained from the Scottish cancer registry. Cases that could not be matched to 1991 census output areas (11) or whose cancer was registered from hospitals outside Grampian or Highland (27) were excluded. The remaining 1960 cases were divided into groups according to health board area of residence, cancer site (lung or colorectal) and distance from a cancer centre (less or more than a straight line distance of 38 km). Using computer-generated random numbers a random sample of 250 cases was selected from each group in Grampian and 100 cases from each group in Highland. In one of the stratified groups in Grampian, there were 248 cases, so all were included. The total sample comprised 1398 cases.

### Clinical data abstraction

Clinical data were abstracted from case notes at teaching and general hospitals in Grampian and Highland by NC and AE using standardized protocols. Disease stage at diagnosis was abstracted or deduced from data in case notes where possible. Case notes from general practitioner-led facilities (general practices and most community hospitals) were not reviewed.

### Outcomes

The main outcome was the presence of disseminated disease at diagnosis, which was defined as distant metastases for colorectal and non-small-cell lung cancers and extensive disease for small cell lung cancer (SIGN, 1997, 1998). Secondary outcomes were emergency presentation to hospital (both cancers), emergency surgery (colorectal cancer only), Dukes stage for colorectal cancer and ISS stage for lung cancer (SIGN, 1997, 1998).

### Main independent variable

The main independent variable was distance to the nearest cancer centre. This rural indicator was selected because it has previously been found to be associated with poorer survival (Campbell et al, 2000). Straight line distances were calculated between patients' place of residence (output area centroids were used – see below) and cancer centres in Aberdeen and Inverness. For analysis, patients were split into 4 groups according to their distance from the nearest cancer centre: 0 to 5 km, 6 to 37 km, 38 to 57 km and ≥58 km. The cutpoints, which approximate to population quartiles for Grampian and Highland, were pre-set on the basis of a previous study (Campbell et al, 2000).

### Additional independent variables

Other variables included in the analysis were settlement size, deprivation, health board of residence, sex, age, smoking status and cancer site. Cases were assigned indices for deprivation, distance to a cancer centre and settlement size based on 1991 census data. Standard area-based indicators (based on postal sectors) have been criticized as insensitive in rural areas where postal sectors cover large areas, and affluence and poverty can coexist in close proximity (Cox, 1998). In order to improve sensitivity, we used the smallest geographical units on which census data were available for Scotland in the 1991 census – output areas. There were 5179 output areas in Grampian and Highland with a median population of 127 (interquartile range 101–164). This use of very small areas units has been found previously to enable inequalities to be shown even in rural areas (Reading et al, 1993; Campbell et al, 2000).

Settlement size was included as a second rural indicator found previously to be associated with health (Weinert and Boik, 1995). Categories were assigned according to the size of conurbation of which each output area was part at the time of the 1991 census. The categories were 100 000 to 1 000 000, 10 000 to 100 000, 1000 to 10 000, 500 to 1000, and <500. Deprivation has been shown to be associated with later stage at diagnosis and poorer cancer survival (Ionescu et al, 1998; Coleman et al, 1999). In this study, deprivation scores were calculated using the method of Carstairs and Morris (1990), but with output areas as the geographical units. The score is based on 4 elements: male unemployment, overcrowded housing, car ownership and low social class. Output areas were divided into 'deprivation' quintiles (each containing approximately one fifth of the population) with the least deprived coded '1' and the most deprived coded '5'.

### Analysis

Data were managed using Microsoft Access version 2 and analysed using SPSS for Windows release 9. In the first instance, outcome data were analysed separately for each cancer. 2 outcomes (disseminated disease at diagnosis and emergency presentation to hospital) were the same for both cancers so, to increase statistical power, data were also analysed on both cancers combined. Outcomes were compared for all categories of the main independent variables using the chi square test. Logistic regression was used to model the main variables and adjust for the additional variables as appropriate.

### RESULTS

Of the stratified random sample of 1398 cases, 4 were found to be duplicates and one did not have cancer. Of the remaining 1393 cases, notes were traced and reviewed for 1323 cases (95%). In terms of the main independent variables and other available data, there were no important differences between cases whose notes were reviewed and those whose notes were not (Table 1). In terms of the main outcomes, data on the presence of disseminated disease were not deducible from cases notes for 67 cases (5%), about half of whom lived more than 58 km from a cancer centre and most of whom were aged 80 years or more. Data on type of referral (emergency or otherwise) were not deducible for 78 cases (6%), but this group had no clinically important differences from the others in the main variables. Data on histological type were present for 512 of 665 lung cancers (77%) – there were no differences in proportions between distance groups.

Table 2 shows that there was a trend for increasing distance from a cancer centre to be associated with increased likelihood of disseminated disease at diagnosis. This trend was statistically significant when data on both cancers were combined. There was no evidence of an association between distance from a cancer centre and emergency admission to hospital or emergency surgery for colorectal cancer.

The unadjusted odds ratio of disseminated disease at diagnosis for patients living 58 km or more from a cancer centre compared to those living within 5 km was 1.47 (Table 3). Other variables which, on univariate testing, had significant relationships were settlement size, deprivation, health board of residence, cancer site, smoking at time of diagnosis and age. When these variables were modelled using logistic regression, only settlement size, health board of residence, and cancer site had significant effects – adjusting for these variables, increasing distance from a cancer centre remained significantly associated with higher chance of disseminated disease at diagnosis (Table 3).

*© 2001 Cancer Research Campaign*

**Table 1** Characteristics of cases included and excluded in the analyses

| | | Case notes not traced (*n* = 10) | Case notes reviewed (*n* = 1323) | Presence of disseminated disease unknown (*n* = 67) | Type of referral unknown (*n* = 78) |
|---|---|---|---|---|---|
| Distance from cancer centre | ⩽5 km | 29 (41) | 421 (32) | 13 (19) | 18 (23) |
| | 6–37 km | 15 (21) | 231 (17) | 6 (9) | 7 (9) |
| | 38–57 km | 13 (19) | 312 (24) | 14 (21) | 26 (33) |
| | ⩾58 km | 13 (19) | 359 (27) | 34 (51) | 27 (35) |
| Settlement size | 100 000–1000 000 | 18 (26) | 360 (27) | 12 (18) | 19 (24) |
| | 10 000–100 000 | 13 (19) | 255 (19) | 8 (12) | 10 (13) |
| | 1000–10 000 | 21 (30) | 430 (32) | 28 (42) | 35 (45) |
| | 500–1000 | 5 (7) | 62 (5) | 8 (12) | 1 (1) |
| | <500 | 13 (19) | 216 (16) | 11 (16) | 13 (17) |
| Deprivation quintile | 1 – least deprived | 12 (17) | 192 (15) | 9 (13) | 15 (19) |
| | 2 | 12 (17) | 233 (18) | 12 (18) | 13 (17) |
| | 3 | 12 (17) | 287 (22) | 16 (24) | 15 (19) |
| | 4 | 22 (31) | 326 (25) | 17 (25) | 17 (22) |
| | 5 – most deprived | 12 (17) | 285 (22) | 13 (19) | 18 (23) |
| Health board of residence | Grampian | 42 (60) | 952 (72) | 55 (82) | 70 (90) |
| | Highland | 28 (40) | 371 (28) | 12 (18) | 8 (10) |
| Sex | Male | 44 (63) | 754 (57) | 40 (60) | 45 (58) |
| Age band | ⩽59 | 17 (24) | 226 (17) | 3 (4) | 16 (21) |
| | 60–69 | 19 (27) | 372 (28) | 6 (9) | 21 (27) |
| | 70–79 | 22 (31) | 468 (35) | 17 (25) | 27 (35) |
| | ⩾80 | 12 (17) | 257 (19) | 41 (61) | 14 (18) |
| Cancer site | Lung | 33 (47) | 665 (50) | 44 (66) | 40 (51) |
| | Colon | 27 (39) | 452 (34) | 15 (22) | 29 (37) |
| | Rectum | 10 (14) | 206 (16) | 8 (12) | 9 (11) |

Values are numbers (percentages) unless otherwise specified.

**Table 2** Numbers (percentages) of cases with disseminated disease at diagnosis, first admitted as emergencies and (for colorectal cancer) requiring emergency surgery

| | | Lung cancer | Colorectal cancer | Both cancers |
|---|---|---|---|---|
| Disseminated disease at diagnosis | | | | |
| Distance from cancer centre | ⩽5 km | 71/215 (33) | 31/193 (16) | 102/408 (25) |
| | 6–37 km | 33/99 (33) | 24/126 (19) | 57/225 (25) |
| | 38–57 km | 48/143 (34) | 27/155 (17) | 75/298 (25) |
| | ⩾58 km | 69/164 (42) | 38/161 (24) | 107/325 (33) |
| *P* value | Global | 0.251 | 0.313 | 0.060 |
| | Trend | 0.098 | 0.112 | 0.031 |
| First admitted as emergency | | | | |
| Distance from cancer centre | ⩽5 km | 78/215 (36) | 68/188 (36) | 146/403 (36) |
| | 6–37 km | 31/98 (31) | 44/126 (35) | 75/224 (33) |
| | 38–57 km | 51/137 (37) | 61/149 (41) | 112/286 (39) |
| | ⩾58 km | 70/175 (40) | 50/157 (32) | 120/332 (36) |
| *P* value | Global | 0.587 | 0.420 | 0.619 |
| | Trend | 0.382 | 0.672 | 0.731 |
| Required emergency surgery | | | | |
| Distance from cancer centre | ⩽5 km | | 29/196 (15) | |
| | 6–37 km | | 14/128 (11) | |
| | 38–57 km | | 29/162 (18) | |
| | ⩾58 km | | 23/172 (13) | |
| *P* value | Global | | 0.388 | |
| | Trend | | 0.906 | |

More detailed data on stage could be deduced from case notes for 1198 (91%) patients (Table 4). Again, more patients who lived further from cancer centres tended to have unstaged disease. Of all 91 patients with small cell lung cancer, 39 (43%) had limited disease at diagnosis, but numbers were too small to draw further conclusions. Of 574 patients with non-small-cell lung cancer, 125 had stage I or II disease (22%), but this proportion was higher for those living within 5 km of cancer centres (56/198, 28%) and lower for those living further away (23/165 (14%)). This trend was statistically significant (*P* value for linear trend = 0.002). After adjusting for other significant variables, the odds ratio of stage I or II disease at diagnosis for patients in the outermost category compared to the innermost one was 0.39 (95% confidence intervals 0.22 to 0.68). There was a similar trend for colorectal cancer. Of all 658 cases, 311 (47%) were Dukes stage A or B at diagnosis, but this varied from 101 out of 196 (52%) patients living within 5 km to 67 out of 172 (39%) cases living more than 58 km away (*P* value for linear trend = 0.035). After adjusting for other significant

 © 2001 Cancer Research Campaign

**Table 3** Odds ratios (95% confidence intervals) for the presence of disseminated disease at diagnosis

| | | Unadjusted odds ratio (95% confidence intervals) | Adjusted odds ratio[a] (95% confidence intervals) |
|---|---|---|---|
| Distance from cancer centre | | | |
| | ⩽5 km | 1 | 1 |
| | 6–37 km | 1.02 (0.70,1.48) | 1.11 (0.63,1.94) |
| | 38–57 km | 1.01 (0.71,1.42) | 1.28 (0.71,2.30) |
| | ⩾58 km | 1.47 (1.07,2.03) | 1.59 (0.91,2.78) |
| *P* value | global | 0.060 | 0.215 |
| | trend | 0.031 | 0.037 |

[a]Adjusted for other variables that remained significant after modelling (settlement size, health board, and cancer site).

variables, the odds ratio of stage A or B disease at diagnosis was 0.62 (95% confidence intervals 0.32 to 1.23) for patients in the outermost group relative to the innermost group.

### DISCUSSION

The main finding of this study was that increasing distance from a cancer centre was associated with a higher chance of disseminated disease at diagnosis. In line with this finding, outlying patients had less chance of limited stage disease at diagnosis. No differences were detected, however, in the proportion of patients requiring emergency admission to hospital or emergency surgery.

### Strengths and limitations

The study benefited from a high rate of case note retrieval (95%) with no evidence of bias between rural and urban groups. The required data were deducible from case notes in nearly all cases, but there was one difference that may have led to bias in the analysis of disseminated disease at diagnosis – cases for whom it was not clear if metastases were present tended to live further from a cancer centre. Unstaged cancers tend to have poorer prognosis than staged cancers (suggesting that they are more advanced), so this bias is likely to have reduced the differences we reported between groups rather than increased it (Merrill et al, 1999; Parry et al, 1999). The study had limited statistical power to analyse each cancer separately so data on both cancers were combined in some analyses. To prevent this causing confounding, the same numbers of cases with each cancer were sampled in each distance group and adjustments were made for cancer site in the analyses. The setting for the study in the north and northeast of Scotland had advantages and disadvantages. With the bulk of hospital and cancer services and about half the population concentrated in 2 locations, and the rest scattered over a large area, it was relatively easy to compare rural and urban differences. On the other hand, it is not possible to determine the reasons for the differences we found or their relevance to areas with different characteristics.

### Relationship to other studies

The findings of the study are consistent with related studies, which were conducted in very different rural areas to those of the north and northeast of Scotland. In a study of colorectal cancer in Calvados (France), Launoy et al (1992) found that women in rural areas were more likely to have metastases at diagnosis than those in urban areas (19% versus 12%). There was, however, no difference in men (18% versus 17%). They also found that rural women were more likely to present with severe clinical symptoms (22% versus 16%). In the United States, Liff et al (1991) reported that rural patients in Georgia were more likely to have unstaged tumours (9% versus 3% for colon; 30% versus 12% for lung) and more non-localized cancers overall (59% versus 54% among whites and 71% versus 64% among blacks). Our findings are also consistent with previous research in Scotland (Campbell et al, 2000). In a survival analysis of cancer registrations, patients who lived further than 38 km from a cancer centre had less chance of firm diagnosis before death (odds ratios of death before diagnosis

**Table 4** Stage at diagnosis

| | Distance from cancer centre | | | |
|---|---|---|---|---|
| | ⩽5 km | 6–37 km | 38–57 km | ⩾58 km |
| Small cell lung cancer | | | | |
| All cases | 27 | 15 | 27 | 22 |
| Limited disease | 13 (48) | 7 (47) | 13 (48) | 6 (27) |
| Extensive disease | 13 (48) | 8 (53) | 14 (52) | 15 (68) |
| Not known | 1 (4) | 0 (0) | 0 (0) | 1 (4) |
| Other lung cancer (ISS stage) | | | | |
| All cases | 198 | 88 | 123 | 165 |
| I | 42 (21) | 16 (18) | 22 (22) | 19 (12) |
| II | 14 (7) | 2 (2) | 6 (5) | 4 (2) |
| III | 59 (30) | 37 (42) | 42 (34) | 59 (36) |
| IV | 57 (29) | 25 (28) | 34 (28) | 54 (33) |
| Not known | 26 (13) | 8 (9) | 19 (15) | 29 (18) |
| Colorectal cancer (Dukes stage) | | | | |
| All cases | 196 | 128 | 162 | 172 |
| A | 17 (9) | 14 (11) | 18 (11) | 12 (7) |
| B | 84 (43) | 47 (37) | 64 (40) | 55 (32) |
| C | 55 (28) | 39 (30) | 42 (26) | 50 (29) |
| D | 31 (16) | 24 (19) | 27 (17) | 38 (22) |
| Not known | 9 (5) | 4 (3) | 11 (7) | 17 (10) |

Values are numbers (percentages within distance category)

*© 2001 Cancer Research Campaign*

were 1.78 for colorectal and 1.14 for lung cancers) and poorer survival after diagnosis (hazard ratios were 1.11 for colorectal and 1.09 for lung cancer).

### Meaning and implications

Our findings suggest that the poorer survival observed in outlying patients in Scotland is at least partly explained by them tending to have more advanced disease at diagnosis. The effect was strongest for patients living more than 58 km (as the crow flies) from the nearest cancer centre (and its associated conurbation) – in Scotland, this comprises about half a million people (10% of the total population). Our study does not explain why this association was present. Distance from cancer centres does not necessarily reflect distance from diagnostic services, at least some of which would be available more locally (in general practices and local hospitals). Difficult access to general services can, however, alter peoples' attitudes – perceived need is inversely related to remoteness (Watt et al, 1993). In a qualitative study, patients with colorectal cancer in remote and rural areas appeared to have lower expectations when evaluating their care, so may delay self-presentation and be more tolerant of delays in referral than their more demanding urban counterparts (Bain and Campbell, 2000). They also reported experiencing more hurdles before reaching specialist care, especially if referred via local non-specialist hospitals.

This study lends further support to the theory that remote and rural patients are disadvantaged in the early diagnosis of cancer. The reasons for this warrant further investigation. In the meantime, remote and rural patients require prompt referral and investigation if they are to benefit from increasingly specialist and centralized cancer services.

### ACKNOWLEDGEMENTS

The study was funded by a Cancer Research Campaign Primary Care Oncology Fellowship. We thank the Scottish Cancer Registry (Information and Statistics Division of the Scottish Health Service) and in particular, Veronica Harris, who extracted the data we sought on Scottish cancer registrations. Thanks to the consultants involved in the care of patients with colorectal and lung cancer in Highland and Grampian Health Board areas, who agreed to the study.

### REFERENCES

Bain NSC and Campbell NC (2000) Treating patients with colorectal cancer in rural and urban areas: a qualitative study of the patients' perspective. *Fam Pract* **17**: 475–479

Campbell NC, Ritchie LD, Cassidy J and Little J (1999) Systematic review of cancer treatment programmes in remote and rural areas. *Br J Cancer* **80**: 1275–1280

Campbell NC, Elliott A, Sharp L, Ritchie LD, Cassidy J and Little J (2000) Rural factors and survival from cancer: analysis of Scottish cancer registrations. *Br J Cancer* **82**: 1863–1866

Carstairs V and Morris R (1990) *Deprivation and health in Scotland*. University Press: Aberdeen

Coleman M, Babb P, Damiecki P, Grosclaude P, Honjo S, Jones J, Knerer G, Pitard A, Quinn M, Sloggett A and De Stavola B (1999) *Cancer survival trends in England and Wales, 1971–95: deprivation and NHS Region*. (*Stud Med Popul Subj* no 61). Stationery Office: London

Cox J (1995) *Rural General Practice in the United Kingdom. Occasional Paper 71*. The Royal College of General Practitioners: London

Cox J (1998) Poverty in rural areas. *BMJ* **316**: 722

Expert Advisory Group on Cancer (1995) *A Policy Framework for commissioning Cancer Services: a report by the Expert Advisory Group on Cancer to the Chief Medical Officers of England and Wales, 1995*. HM Stationery Office: London

Information and Statistics Division, National Health Service in Scotland (1995) *Scottish Health Service Costs. Year ending 31st March, 1995*. ISD Publications: Edinburgh.

Information and Statistics Division, National Health Service in Scotland (1999) *Scottish Health Statistics 1998*. ISD Publications: Edinburgh.

Ionescu MV, Carey F, Tait IS and Steele RJC (1998) Socioeconomic status and stage at presentation of colorectal cancer. *Lancet* **352**: 1439

Launoy G, Le Coutour X, Gignoux M, Pottier D and Dugleux G (1992) Influence of rural environment on diagnosis, treatment and prognosis of colorectal cancer. *J Epidemiol Community Health* **46**: 365–367

Liff JM, Chow WH and Greenberg RS (1991) Rural-urban differences in stage at diagnosis. Possible relationship to cancer screening. *Cancer* **67**: 1454–1459

Merrill RM, Henson DE and Barnes M (1999) Conditional survival among patients with carcinoma of the lung. *Chest* **116**: 697–703

Parry JM, Collins S, Mathers J, Scott NA and Woodman CB (1999) Influence of volume of work on the outcome of treatment for patients with colorectal cancer. *Br J Surg* **86**: 475–481

Reading R, Jarvis S and Openshaw S (1993) Measurement of social inequalities in health and use of health services among children in Northumberland. *Arch Dis Child* **68**: 626–631

Scottish Intercollegiate Guidelines Network, Scottish Cancer Therapy Network (1997) *Colorectal Cancer* SIGN: Edinburgh

Scottish Intercollegiate Guidelines Network, Scottish Cancer Therapy Network (1998) *Management of Lung Cancer*. SIGN: Edinburgh

Selby P, Gillis C and Haward R (1996) Benefits from specialised cancer care. *Lancet* **348**: 313–318

Summerton N (1999) *Diagnosing cancer in primary care*. Radcliffe: Oxford

Watt IS, Franks AJ and Sheldon TA (1993) Rural health and health care. *BMJ* **306**: 1358–1359

Weinert C and Boik J (1995) MSU rurality index: development and evaluation. *Research Nurs Health* **18**: 453–464

*© 2001 Cancer Research Campaign*
